# Blood Eosinophil Count in Asthma Is Associated With Increased Abdominal Aortic Diameter and Increased Vascular Stiffness

**DOI:** 10.2147/JAA.S483504

**Published:** 2025-02-20

**Authors:** Leonie Biener, Andrea Budimovska, Dirk Skowasch, Carmen Pizarro, Ben Christoph Frisch, Georg Nickenig, Max Jonathan Stumpf, Christian A Schaefer, Nadjib Schahab

**Affiliations:** 1 Department of Internal Medicine II - Cardiology, Pneumology and Angiology, University of Bonn, Bonn, Germany

**Keywords:** blood eosinophil count, asthma, abdominal aortic diameter, aneurysm, vascular stiffness

## Abstract

**Background:**

Asthma is associated with atherosclerosis and abdominal aortic aneurysm (AAA). However, the underlying pathomechanisms remain elusive. Blood eosinophil count (BEC) is implicated in both eosinophilic asthma and arterial wall inflammation.

**Objective:**

To explore the possible association of BEC in asthma and abdominal aortic artery changes.

**Methods:**

112 outpatients were prospectively enrolled in this exploratory study. Abdominal aortic diameter was measured using ultrasonography imaging, while vascular speckle tracking was utilized to evaluate vascular strains. Patients were stratified into two groups, with n=66 patients with a BEC of ≥300 n/µL and n=46 patients with <300 n/µL. Both groups exhibited no significant disparities in cardiovascular risk factors; however, the high BEC group was more frequently male.

**Results:**

The aortic diameter was wider in patients with a BEC ≥300 n/µL (1.46 ± 0.25 cm vs 1.67 ± 0.63 cm, p=0.018). Three patients were diagnosed with an AAA, all had a BEC ≥300 n/µL. Patients with a BEC ≥300 n/µL exhibited lower strain values, indicative of higher vascular stiffness, including radial strain (2.65 ± 1.38% vs 4.46 ± 2.59%; p<0.001). BEC exhibited a positive correlation with abdominal aortic diameter (R²=0.131, b=0.000, p<0.001), and a negative correlation with radial strain values (R²=0.131, b=−0.002, p=0.001) in sex-adjusted linear regression.

**Conclusion:**

In patients with asthma, blood eosinophil count (BEC) is correlated with a wider aortic diameter and heightened vascular stiffness in the abdominal aorta. Hence, they may be at an elevated risk of developing an AAA.

## Introduction

Asthma is a respiratory condition characterized by airway inflammation and increased bronchial reactivity, as delineated in the 2023 German national Asthma Guidelines.^1^ Asthma is progressively acknowledged as a heterogeneous condition with various phenotypes, yet eosinophilic asthma lacks clearly defined diagnostic thresholds. According to the German guideline, the diagnosis of eosinophilic asthma necessitates at least two instances of detecting a minimum of 300 eosinophil granulocytes/µL outside of exacerbations.^1^ However, the GINA Main Report 2023 does not delineate a specific threshold but recommends contemplating type 2 inflammation when blood eosinophils are ≥ 150 n/µL.^2^

The aortic aneurysm, characterized by the bulging of the aortic wall, frequently presents asymptomatically. Nevertheless, its acute clinical manifestations, including rupture and dissection, carry a serious prognosis, with a mortality rate of 90% outside the hospital and over 40% in-hospital mortality.^3^ Inflammation has previously been associated with aortic dissection, with immune cells, matrix proteins, cytokines, and chemokines all contributing to the pathomechanism.^4^ Moreover, asthma has been linked to systemic inflammation, increased arterial stiffness and atherosclerosis.^5–7^ Studies have also indicated a potential correlation between the count of differential leukocytes and abdominal aortic aneurysm, including eosinophils.^8^ The underlying mechanism remains unclear. However, the collective of patients with eosinophilic asthma has not been investigated with regard to vascular pathologies. Therefore, our approach was to analyse the vascular status of asthma patients in terms of the abdominal aortic diameter and arterial stiffness with regard to their eosinophil count.

## Materials and Methods

### Study Design

This cross-sectional study was conducted at the Outpatient Department of Pneumology and Angiology, University Hospital of Bonn, between June 2022 and February 2023. A total of 112 consecutive patients aged 18 years and older with pre-existing asthma were enrolled. Exclusion criteria encompassed lack of consent or inability to consent to the study, as well as a BEC of ≥1500 cells/µL to exclude eosinophilia associated with comorbidities such as hypereosinophilic syndrome and eosinophilic granulomatosis with polyangiitis. None of the patients presented with an acute asthma exacerbation during the examination. Patient history, current medication, and eosinophil levels were obtained from patient records. Pulmonary function tests were performed as part of the clinical routine at the day of study inclusion. The study exclusively incorporated color-coded duplex sonography as an additional examination, which is elaborated upon below. All patients provided written informed consent, and authorization was granted by the local ethics committee (Medical Faculty of the University of Bonn, Rheinische Friedrich-Wilhelms-Universität Bonn, No 117/22). The study was conducted in accordance with the principles outlined in the Declaration of Helsinki.

### Vascular Assessments

The vascular examination took place at the day of study inclusion during a routine check-up performed by experienced examiners who were blinded to the results of blood eosinophil count. The examination included B-mode and color-coded duplex sonography of the abdominal aorta (AA), along with the measurement of the diameter of the abdominal aorta using the leading-to-leading edge method, using Philips^®^ iE33^®^ (Hamburg, Germany).^9^ Sonographic assessment of the abdominal aorta (AA) is a cost-effective and precise method for screening and diagnosing aortic aneurysms, demonstrating high sensitivity (94–100%) and specificity (98–100%). An abdominal aortic aneurysm (AAA) was defined as a vessel diameter greater than or equal to 3.0 cm, according to the ESC guidelines on aortic diseases as well as the S3 guideline on AAA of the German Society for Vascular Surgery and Vascular Medicine.^10^,^11^

**Figure 1:**
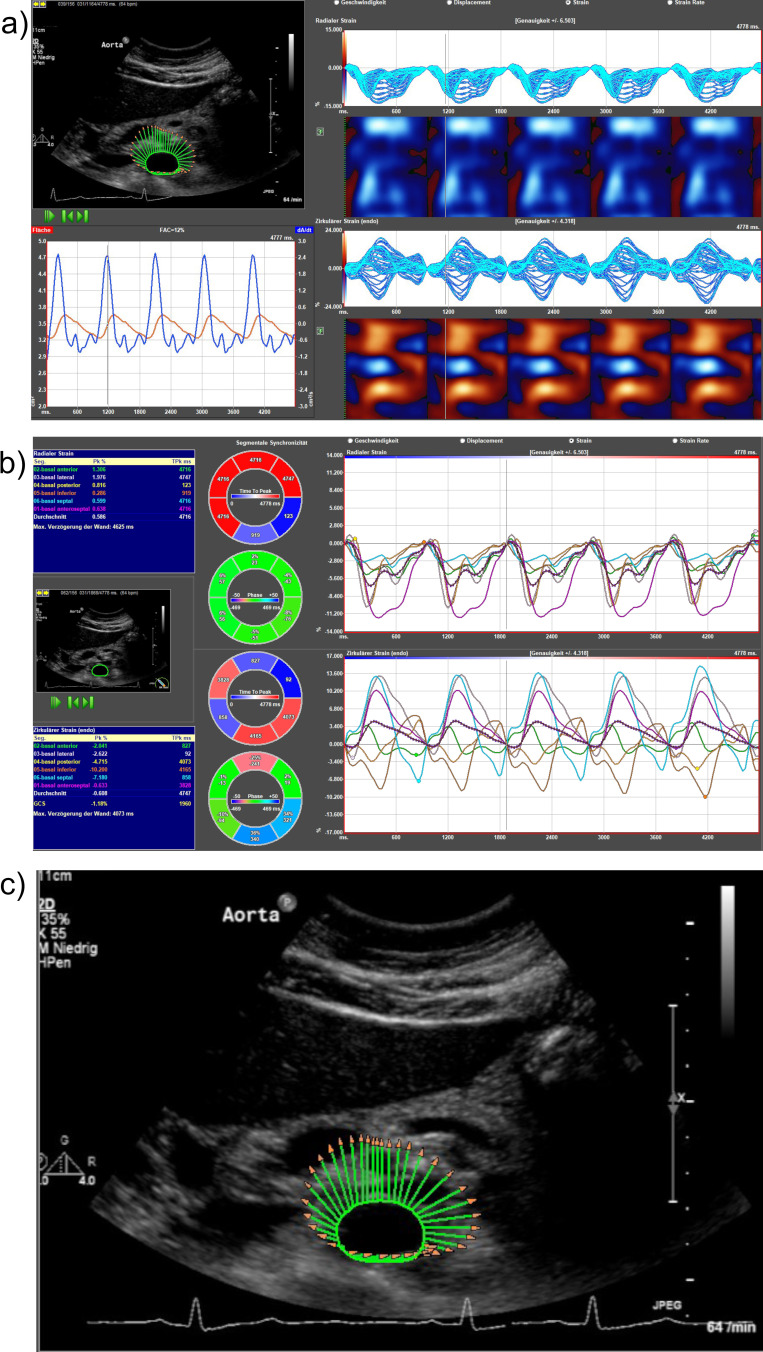
Strain analysis performed using ImageArena Version 4.6, TomTec Systems. (**a** and **b**) Graphic display of the radial strain (top) and the circumferential strain (bottom) by the software. (**c**) The green-shaded region surrounding the abdominal aorta indicates the radial expansion of the aortic wall in %.^6^

Additionally, a B-mode recording of the vessel cross-sectional area during five cardiac cycles was captured, triggered by electrocardiography (ECG). Subsequently, strain analysis was performed using ImageArena Version 4.6 software from TomTec Systems GmbH in Munich, Germany, following the previously described methodology.^9^,^12^,^13^ The strain analysis includes various parameters, such as radial strain (radial expansion of the vessel during one cardiac cycle in %), circumferential strain (change of the vessel wall circumference during one heart cycle in %), radial strain rate and circumferential strain rate (dynamic parameters of the vessel wall motion over time (1/s)), radial displacement (overall movement of the vessel wall in millimeter (mm)) and radial velocity in centimeters per second (cm/s). Figure 1 provides an illustration of a strain analysis. Lower strain values indicate higher vascular rigidity.^14^

Our primary outcome measures were defined as the aortic diameter and radial strain.

### Blood Parameters

The count of eosinophil granulocytes from EDTA-blood and serum levels of Immunoglobulin E (IgE) were evaluated using the latest patients’ medical records. In order to enhance accuracy and reliability we included patients with at least two documented elevated blood eosinophil levels within the last 12 months. The most recent eosinophil count obtained outside of an acute exacerbation and without systemic corticosteroid treatment or biologic therapy such as anti-IL-5(R), anti-IL-4R, or anti-IgE, was recorded.^15^,^16^

### Asthma

Results from the Asthma Control Test (ACT) were obtained to evaluate disease control. The questionnaire consists of five questions, yielding a scale ranging from five to 25 points. Higher scores indicate better disease control. The minimum clinically important difference (MCID) is 3 points.^17^ Furthermore, fractional exhaled nitric oxide (FeNO) values were recorded, as it is routinely assessed, serves as a marker of type 2 inflammation according to GINA guidelines, and correlates with disease activity.^2^,^18^ Elevated NO levels contribute to bronchial hyperreactivity, airway inflammation, and tissue damage, and have also been linked to aneurysm formation in mice models.^19^,^20^ For both values we used the most recent from patients medical records, outside of an acute exacerbation.

### Statistical Analysis

Data collection and analysis were conducted using IBM^®^ SPSS^®^ Statistics, Version 29.0. Figures were created with GraphPad Prism 10.1.1 (GraphPad Software, Inc., Boston, MA, USA). Continuous data are presented as mean ± standard deviation (SD), while categorical data are expressed as n (%). To compare two groups for continuous variables, the unpaired Student’s *t*-test was utilized. In cases where the Levene test indicated heterogeneity of variances, the Welch test was applied. For non-parametric variables, the Chi-squared test or Fisher’s exact test were employed, as appropriate. Linear regression analysis was conducted to assess the correlation between two metric variables. Linear regression concerning vascular parameters was adjusted by sex category. Results with a p-value (two-sided tests) of less than 0.05 were deemed statistically significant. Not all variables could be fully determined for all patients; missing values are indicated in the tables and figures.

## Results

### Patients Characteristics

We included 112 patients, of whom 42 (37.5%) were male. Patients were stratified based on their blood eosinophil count. One group consisted of 66 patients with a BEC ≥300 n/µL, while the other group comprised 46 patients with a BEC <300 n/µL. There were no significant differences observed in age, body mass index (BMI), number of pack years (PY), hypercholesterolemia, arterial hypertension, family history of myocardial infarction or stroke, fractional exhaled nitric oxide (FeNO), and years since asthma diagnosis between the two groups.

Patients with a BEC ≥300 n/µL demonstrated poorer asthma control, as assessed by the Asthma Control Test (ACT) score (16.1 ± 5.3 vs 13.4 ± 5.7, p=0.014). Additionally, individuals in the high BEC group were more frequently male (23.1% vs 45.5%, p=0.037) and received biologic therapy more often (32.6% vs 69.7%, p<0.001). Furthermore, lung function, measured by forced expiratory volume in 1 second (FEV1) % predicted, was significantly lower in patients with ≥300 Eos/µL (77.6% ± 18.6 vs 65.0 ± 19.5, p<0.001), and they experienced a higher rate of acute exacerbations in the last year (1.4 ± 2.1 n/year vs 2.7 ± 2.4 n/year, p=0.014). Accordingly, the proportion of severe asthma was higher among those with BEC ≥300 n/µL (24.3% vs 89.4%). All baseline characteristics are presented in Table 1.

**Table 1:** Baseline Characteristics

Variables	n	BEC <300 n/µL n=46	BEC ≥300 n/µL n=66	p-value
Sex, male	112	12 (26.1)	30 (45.5)	0.037*^a^
Age (years)	112	52.8 ± 16.5	58.2 ± 13.6	0.061^b^
BMI (kg/m^2^)	112	26.2 ± 4.5	27.7 ± 6.5	0.194 ^b^
Hypercholesterolemia	112	13 (28.3)	20 (30.3)	0.816 ^a^
Diabetes mellitus	112	3 (6.5)	7 (10.6)	0.552 ^c^
Arterial Hypertension	112	16 (34.8)	33 (50.0)	0.110 ^a^
Smoking: never	110	25 (54.3)	36 (54.5)	0.986^a^
Smoking: former (PY)	110	6,7 ± 11.6	7,4 ± 15.2	0.800^b^
Smoking: current (PY)	110	1,7 ± 7.0	1,2 ± 7.7	0.732^b^
ACT Score	105	16.1 ± 5.3	13.4 ± 5.7	0.014* ^b^
Exacerbations/year	111	1.4 ± 2.1	2.7 ±2.4	0.004* ^b^
Years since asthma diagnosis	111	24.3 ± 17.1	23.8 ± 17.7	0.890 ^b^
Family history for MI/stroke	112	14 (30.4)	17 (25.8)	0.586 ^a^
FeNO (ppb)	84	34.6 ± 44.2	42.5 ± 38.4	0.389 ^b^
BEC (n/µL)	112	151.0 ± 67.6	587.4 ± 259.7	<0.001* ^d^
IgE (U/mL)	106	444.7 ± 650.7	524.7 ± 1027.5	0.655 ^b^
FeV_1_ (% predicted)	112	77.6 ± 18.6	65.0 ± 19.5	0.001* ^b^
Biologic therapy	112	15 (32.6)	46 (69.7)	<0.001* ^a^
Severity	112			<0.001* ^a^
- mild/moderate Asthma		21 (45.7)	7(10.6)	
- severe Asthma		25(54.3)	59(89.4)	

**Notes**: Data are presented as mean ± standard deviation or n (%). *= p<0.05 with values indicated in bold express statistically significant results. ^a^Chi-square-test. ^b^Unpaired *t*-test. ^c^ Fisher’s exact test. ^d^ Welch test.

**Abbreviations**: ACT, asthma control test; BEC, blood eosinophil count; BMI, body mass index; FEV1, forced expiratory Volume in 1 second; FeNO, fractional exhaled Nitric Oxide; IgE, Immunoglobulin E; MI, myocardial infarction; PY, pack-years.

### Aortic Diameter

**Figure 2:**
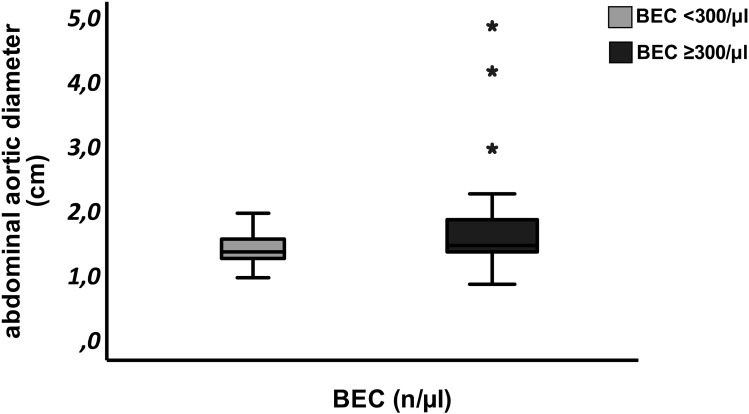
The abdominal aortic diameter was compared between asthma patients with blood eosinophil count (BEC) <300/µL and those with BEC ≥300/µL. Patients with BEC ≥300/µL exhibited a significantly greater aortic diameter compared to those with BEC<300/µL (1.46 ± 0.25 cm vs 1.67 ± 0.63 cm; p=0.018). Outliers are denoted by asterisks (*).^9^

As a primary outcome, our data revealed a significantly wider aortic diameter in patients with a BEC ≥300 n/µL compared to those with <300 n/µL (1.46 ± 0.25 cm vs 1.67 ± 0.63 cm, p=0.018), as illustrated in Figure 2. Three patients diagnosed with an abdominal aortic aneurysm proceeded for further clinical examination, notably all three patients had a BEC of ≥300 n/µL.

**Figure 3:**
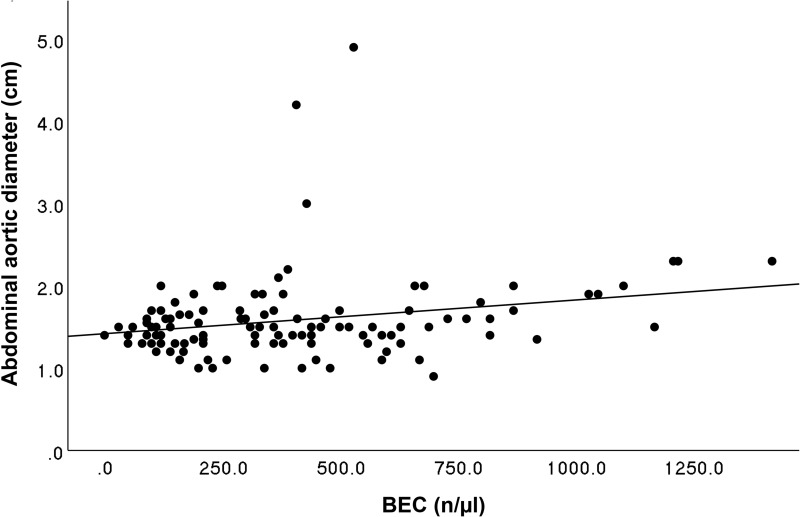
Linear regression analysis of the blood eosinophil count (BEC) and the abdominal aortic diameter, adjusted for sex category. The results demonstrate a small but significant positive correlation between BEC and the aortic diameter *(R²* = 0.131, b = 0.000, p < 0.001).^9^

Considering that the gender is an independent risk factor for AAA and more male patients with BEC ≥300 n/µL than BEC<300 were included, we adjusted the linear regression for sex category. It revealed a weak but significant positive correlation between BEC and the aortic diameter (R² = 0.131, b = 0.000, p<0.001), as depicted in Figure 3.

### Strain Analysis

Vascular strain analysis demonstrated reduced arterial elasticity in patients with BEC ≥300 n/µL compared to those with BEC <300 n/µL. The primary outcome, radial strain, was significantly lower in the group with BEC ≥300 n/µL (2.65 ± 1.38% vs 4.46 ± 2.59%; p<0.001), suggesting potential (pre-) atherosclerotic artery changes.

**Figure 4:**
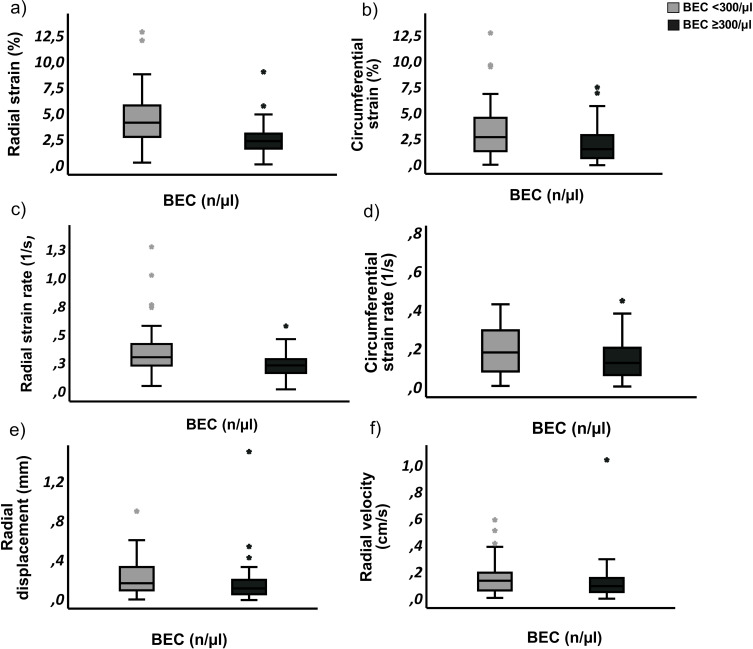
Analysis of the mean strain parameters between two patient groups stratified by blood eosinophil counts (BEC) <300 n/µL and BEC≥300 n/µL. Patients with BEC ≥300 n/µL group showed significantly reduced strain values in multiple measures: (**a**) radial strain (p < 0.001), (**b**) circumferential strain (p = 0.01), (**c**) radial strain rate (p < 0.001), and (**d**) circumferential strain rate (p = 0.009). No statistically significant differences were observed for radial velocity (**e**) or radial displacement (**f**). Outliers are denoted by asterisks (*).^10^

Furthermore, patients with high BEC showed lower values of circumferential strain (2.15 ± 1.79% vs 3.35 ± 2.68%, p=0.01), radial strain rate (0.24 ± 0.10 1/s vs 0.38 ± 0.23 1/s; p<0.001) and circumferential strain rate (0.15 ± 0.10 1/s vs 0.23 ± 0.18 1/s; p= 0.009), as depicted in Figure 4.

No significant difference between the groups was observed regarding radial displacement (0.17 ± 0.21 mm vs 0.23 ± 0.18 mm; p=0.097) and radial velocity (0.13 ± 0.14 cm/s vs 0.16 ± 0.13 cm/s; p=0.208), as illustrated in Figure 4.

**Figure 5:**
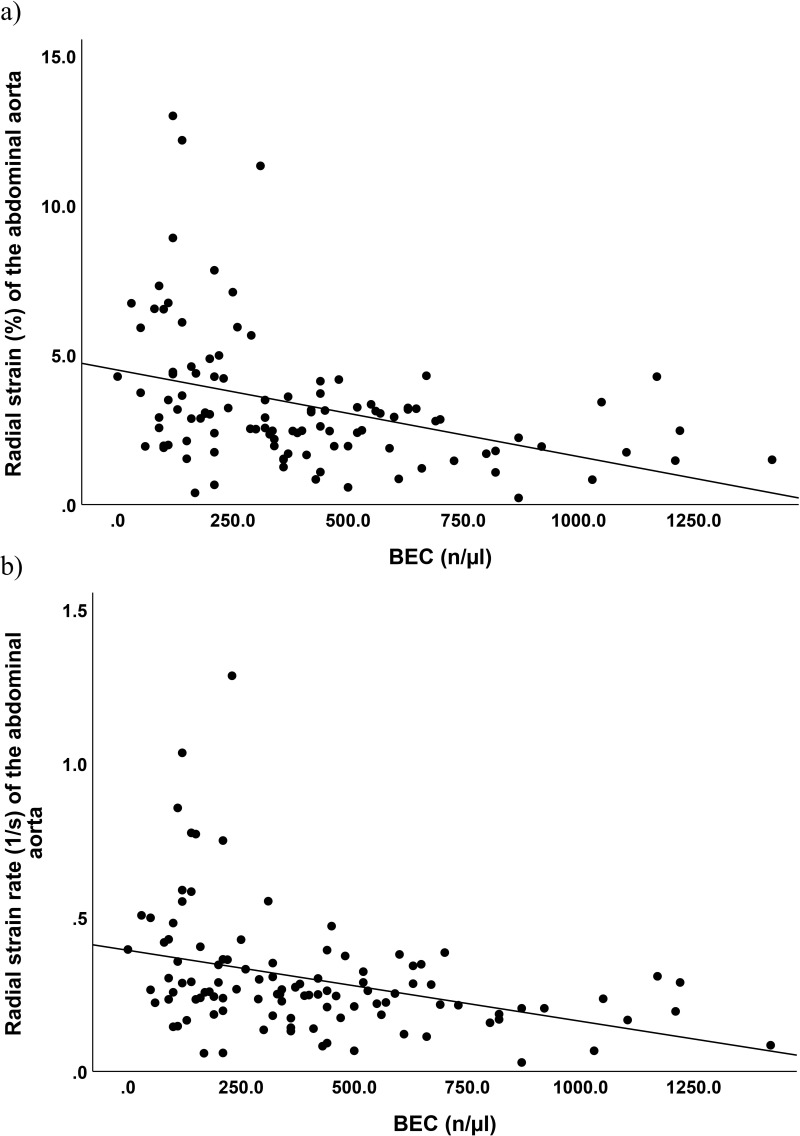
Linear regression analysis of blood eosinophil count (BEC) and abdominal aortic strain (rate), adjusted for sex category. The analysis reveals a small but significant negative correlation between BEC and (**a**) the radial strain *(R²* = 0.131, b = −0.002, p = 0.001), and (**b**) the strain rate *(R²* = 0.327, b = −0.000, p = 0.003) (**b**).^10^

Linear regression analysis adjusted for sex category revealed a weak but significant negative correlation of BEC and the radial strain (R²=0.131, b = −0.002, p = 0.001) and radial strain rate of the AA (R² = 0.327, b = −0.000, p = 0.003), shown in Figure 5.

### FeNO and ACT

FeNO and ACT as parameters reflecting disease activity and disease control, did not correlate with a wider aortic diameter (p=0.419 for FeNO; p=0.815 for ACT) or radial strain (p=0.095 for FeNO, p=0.189 for ACT) in linear regression analysis.

## Discussion

Asthma has been linked to an elevated cardiovascular risk, atherosclerotic artery changes, and the development of aortic aneurysms and ruptures.^6^,^21^,^22^ Despite systemic inflammation being considered a common pathophysiological factor, the precise mechanisms remain incompletely understood. Inflammation markers such as Interleukin-6 (IL-6), C-reactive protein (CRP), and others are associated with both increased asthma exacerbations and cardiovascular events.^23^,^24^ The role of type 2 inflammation in atherosclerosis is garnering attention.^25^ Consequently, eosinophil granulocytes, as effectors of the type 2 inflammation cascade, have become the focus in several cardiovascular studies.

Our study revealed a significantly wider aortic diameter in asthmatic patients with a BEC ≥300 n/µL, and all three patients with newly diagnosed AAA had a high BEC. This finding is consistent with previous research. A Danish register study reported an elevated risk of AAA in patients with asthma, although eosinophil quantity was not specified.^22^ Similarly, a large cohort study conducted in the United States, involving over 11.000 patients, demonstrated an association between increased eosinophils and AAA. Patients with AAA exhibited significantly higher eosinophil levels.^8^ It is noteworthy that this study did not specifically target patients with asthma.

However, it is not only an epidemiological association between eosinophils and aortic aneurysms that has been demonstrated. In our study, we were able to illustrate a modest yet statistically significant correlation between blood eosinophil count and aortic diameter. While Type 2 dominant inflammation has not been identified in early atherosclerotic lesions, it has been recognized in aortic aneurysms.^26^ These findings are not necessarily conflicting. On one hand, there is discussion regarding the potential involvement of eosinophils in thrombotic processes, which are more prevalent in later stages such as aneurysms.^22^ On the other hand, according to Marx et al, eosinophils may contribute to atherosclerosis development without infiltrating the vascular wall and therefore may not be present in the earlier atherosclerotic lesions themselves.^27^

Aortic aneurysms commonly present after the age of 65,^28^ demonstrating the importance of exploring earlier atherosclerotic lesions in our research inquiry. Our examination of vascular strains as indicators of early atherosclerotic changes unveiled heightened vascular stiffness of the abdominal aorta in asthma patients with elevated eosinophil levels. The influence of eosinophils on vascular stiffness in linear regression was modest, as anticipated, considering that atherosclerosis involves multiple contributing factors. Given the complex nature of atherosclerosis, minor alterations in blood eosinophil count are unlikely to exert a significant direct impact on aortic diameter or vascular stiffness.

- Previous studies have consistently shown a correlation between allergic asthma and atherosclerotic vascular changes, including reduced strain values.^6^,^7^ An earlier discovery from our research team, suggesting that asthma severity correlates with more significant vascular changes, lends support to the hypothesis that systemic inflammation plays a pivotal role. The asthma patients included in our study exhibited elevated IgE levels, which are also indicative of type 2 inflammation.^6^ It is essential to mention that in our study, the patient group with high eosinophils demonstrated a greater number of exacerbations, severe asthma, and a higher utilization of biologic therapy. This aligns with the typical clinical presentation of eosinophilic asthma, which is inherently associated with a more severe disease course.^29^ In our patient population, disease activity or severity at time of inclusion, as measured by FeNO and ACT, did not exhibit an association with the artery changes. Nevertheless, patients with higher BEC did have more pronounced disease, and a higher burden of asthmatic eosinophil inflammation over time. Our study suggests BEC as connection between asthma and artery changes, but other inflammation markers may have had a synergistic effect. Furthermore, an effect of asthma therapy cannot be ruled out either, whereby oral corticosteroids were associated with accelerated atherosclerosis, while a protective effect was presumably suggested by anti-inflammation under ICS.^30^ So far there is no sufficient evidence for a proatherosclerotic effect of IL5-targeted therapy.^31^

Although these findings are encouraging, mouse models have not yet convincingly demonstrated the influence of eosinophils on aortic aneurysm formation. Liu et al demonstrated that allergic lung inflammation exacerbates angiotensin II–induced abdominal aortic aneurysm formation in mice, whereas anti-IgE-targeted therapy suppresses AAA formation.^32^ Conversely, other models have suggested a protective role of eosinophils and type 2 innate cells in AAA development.^33^,^34^ One study reported that mice deficient in eosinophils exhibited fewer atherosclerotic lesions, while another study found that the absence of eosinophils had no impact on atherosclerosis development.^27^,^35^

The findings suggest that the relationship between eosinophils and atherosclerosis, as well as the formation of abdominal aortic aneurysms, is intricate and not yet fully elucidated. Interestingly, Parikh et al have also reported an increased hazard ratio for the presence of AAA with low eosinophil counts.^8^ This implies that different pathomechanisms may contribute to AAA genesis at both low and high eosinophil counts, potentially in different stages from early atherosclerosis to AAA and rupture. However, further research is needed to gain a more comprehensive understanding of these relationships, particularly in asthma patients.

In summary, our study revealed an association between eosinophil count and heightened abdominal arterial stiffness and larger abdominal aortic width in patients with asthma. Therefore, these patients may be at an elevated risk of developing an abdominal aortic aneurysm. Further research is needed for confirmation of our hypothesis.

## Limitations

Due to the exploratory nature of the study, only hypotheses can be generated, and no causal relationships can be established. There is a selection bias as the study was conducted at a single center in a university hospital, implying that a potentially sicker patient population was examined; however, this also applies to the control group. Additionally, while there are official diagnostic criteria for eosinophilic asthma, the eosinophil count can vary significantly. A single measurement may not always accurately reflect the phenotype. Therefore, we have assessed two eosinophil counts and recorded the most recent one, unaffected by oral steroid therapy and obtained outside of exacerbations to present the most precise depiction possible.

Given that the development of an aortic aneurysm is a process spanning several years and the risk of AAA increases with age, while asthma patients are often younger, the age of our cohort is relatively young. Consequently, we also examined pre-atherosclerotic changes. Furthermore, there exists an alternative definition of an aneurysm, denoting an expansion greater than 150% of the expected diameter based on gender, age, and body size.^36^ It is conceivable that with an alternative AAA definition, different results might have been obtained; however, we do not anticipate significant differences in the outcomes.
